# Putting meat on the bone: how to fast-track innovative medicines to those who need them and generate data to justify continued use

**DOI:** 10.1093/haschl/qxae095

**Published:** 2024-08-09

**Authors:** Daniel Ollendorf, Christopher Henshall, Marie Phillips, Patricia Synnott, Lloyd Sansom, Sean Tunis

**Affiliations:** Center for the Evaluation of Value and Risk in Health, Tufts Medical Center, Boston, MA 02111, United States; Institute for Clinical and Economic Review, Boston, MA 02108, United States; Office of Health Economics, London SE1 2HB, United Kingdom; Center for the Evaluation of Value and Risk in Health, Tufts Medical Center, Boston, MA 02111, United States; Center for the Evaluation of Value and Risk in Health, Tufts Medical Center, Boston, MA 02111, United States; University of South Australia, Kent Town, South Australia 5071, Australia; Center for the Evaluation of Value and Risk in Health, Tufts Medical Center, Boston, MA 02111, United States; Rubix Health, LLC, Baltimore, MD 21210, United States

**Keywords:** drug approvals, FDA, health technology assessment (HTA), regulatory approval, market authorization

## Abstract

Regulatory agencies worldwide have taken significant steps to expedite approval and market authorization of medicines based on their potential to address areas of significant unmet medical need and severe disease burden. However, initial approval of such medicines is often accompanied by limited evidence of benefit, posing a conundrum for payers and health systems who may desire greater certainty of their value. This paper describes a system of “accelerated access” to manage these tensions and coordinate activities across stakeholders, based on discussions held at a multi-stakeholder convening in June 2023. We focus on 6 core, near-term actions that can be taken to improve the current system: clarifying criteria for expedited regulatory approval, enhancing stakeholder coordination, creating expedited pathways in payer and health technology assessment settings, developing joint regulatory/payer/health technology assessment guidance on study design and data needs, linking pricing policy to data uncertainty, and improving patient and public understanding of the processes involved as well as the risks and benefits of the relevant medicines. Many of these actions will require additional resources and personnel, and some will necessitate unprecedented levels of coordination. Nevertheless, each action is designed to work with minimal adjustments to the current system rather than demanding an entirely new approach.

## Introduction

Over the past 30 years, the US Food and Drug Administration (FDA), European Medicines Agency (EMA), and other regulatory agencies worldwide have established systems and processes designed to speed clinical development and review of medicines that promise to cure severe and intractable diseases or otherwise extend and improve the quality of life for individuals suffering from them.^1^ Approval under these pathways is known by several names, including accelerated, conditional, early, and provisional, depending on what part of the world you are in.

The descriptors themselves highlight both the principles underlying the pathways and the tensions they generate. They are called “accelerated” or “early” because of the perceived urgent need to save or greatly improve lives that are threatened or diminished, with a corresponding expected loss of life and/or health associated with a slower, traditional approval pathway. They are also known as “conditional” or “provisional” due to the understanding that such medicines will initially be approved based on their promise, which will ultimately need to be confirmed by clear demonstration of benefit for the medicine to remain available. These key considerations are often directly in conflict, with drug developers and patient advocates requesting immediate access based on preliminary data and perceived urgency, while health technology assessment (HTA) agencies and payers may desire greater certainty about whether the medicine will fully deliver on expectations. The result is that, while regulators may give rapid market authorization to a new medicine through an expedited pathway, patient access—which, for most patients, depends on payer approval—may take considerably longer. Whether the additional time taken in payer review causes or prevents harm to patients cannot be known with high certainty until confirmatory studies are completed.

A multi-stakeholder discussion was convened in Adelaide, Australia, in June 2023 to discuss the regulatory pathways currently in place, the challenges they present with access and evidence, and the responsibilities that each stakeholder has if we are to move from a focus on expedited regulatory approval to a fully coordinated system of accelerated access (AA) that could promote rapid and appropriate patient access, optimal patient outcomes, health system sustainability, and efficient innovation. The meeting was organized by Bellberry Ltd, a not-for-profit organization that promotes the welfare of research participants and the quality, efficiency, and effectiveness of research.^2^ A full report of this meeting and a statement of principles are available for public review^3,4^; the meeting proceedings have also been summarized in a peer-reviewed publication.^5^ The purpose of this follow-on paper is to extend the thinking beyond principles and into actionable, near-term steps that could be taken to improve the system. We have identified 6 core actions (see Table 1) that key parties could take, separately and together, to implement the crucial elements of a coordinated accelerated access system, along with key feasibility considerations for each.

**Table 1.** Core actions toward a coordinated accelerated access system.

Type of action	Feasibility issues
Make Criteria for Expedited Regulatory Approval Clear Empirical data for unmet need and disease severity Prespecified thresholds for qualification Framework to link surrogate endpoints with potential benefit	Agreeing and defending thresholds Messaging to patient advocates
Enhance Coordination Between Key Stakeholders Scientific dialogue between all relevant parties Quarterly regulator–HTA body communication Legal scrutiny of limitations on communications Common warehouse for clinical trial and real-world data HTA observation of manufacturer–regulator discussions Opportunities for patient and caregiver engagement	Data infrastructure Agency coordination Resources and personnel
Create an Accelerated Pathway in HTA and Payer Settings Distinct accelerated pathway for appraisal Unique process to address urgency and uncertainty Early HTA body–manufacturer conversations Outline expectations for all stakeholders Distinct requirements for manufacturers	Resources and personnel Mitigating “decision” uncertainty
Develop Joint Guidance on Study Design and Data Needs Early scientific advice from regulators and HTA bodies Outcome selection Measurement approaches Study design Data collection	Resources and personnel Agency coordination
Link Pricing Policy to Data Uncertainty Pricing mechanism that responds to outcomes Managed entry agreements Risk-sharing mechanisms Price increase with reduced uncertainty Initial prices linked to comparators/alternatives	Resources and personnel Data requirements Acceptance of methods Integration with local policies
Improving Key Stakeholder Understanding of and Support for AA Drugs and Processes Clear articulation of benefits and risks Plan for confirmation of benefit Lay-friendly clinical summaries Public commentary on draft decisions	Resources and personnel Agency coordination

Abbreviations: AA, accelerated access; HTA, health technology assessment.

## Make criteria for expedited regulatory approval clear

One challenge with the current regulatory programs that offer expedited approval is a lack of clear criteria for qualification. The FDA and EMA list both unmet medical need and the presence of a serious, debilitating, or life-threatening condition as pathway criteria,^6,7^ but provide no additional detail or context. While unmet need and disease severity currently involve qualitative judgments, these could be informed by empirical data. For example, the Global Burden of Disease study provides country- and disease-specific estimates of current burden based on the disability-adjusted life-year (DALY), a standardized measure of the impact of disease on disability and premature mortality.^8^ Other countries routinely measure the quality-adjusted life-year (QALY) “shortfall,” an estimate of the gap in quality of life and life expectancy among those with vs without a given disease.^9^ Such measures could be compared with a prespecified threshold that represents a significant enough unmet need for an expedited pathway to be triggered.

Another gray area for these pathways is the determination of when an unvalidated surrogate endpoint is associated with potential clinical benefit, which may come into play when key outcomes are truly unknown (eg, very rare diseases) or when relationships vary by subtype of a given disease (eg, progression-free survival is correlated with overall survival in some cancers but not others). Taking the time necessary to validate such surrogates clearly defeats the purpose of expedited approval; it is feasible, however, to use reasonable criteria to determine whether a given surrogate endpoint is likely to predict clinical benefit. Ciani and colleagues^10^ developed a useful hierarchy for considering the level of clinical or biological correlation between surrogate and critical outcomes, with specific considerations for (1) strength of association and (2) a threshold for benefit in the surrogate that would generate meaningful clinical improvement. This sort of framework could provide useful guidance for determinations of when a specific surrogate should be considered “reasonably likely” to predict meaningful clinical benefit.

While the approaches described above would help to explain decisions and improve consistency (and hence confidence in the agencies involved), messaging on their use will have to be carefully constructed to both defend the thresholds that will trigger an expedited pathway and explain to patients and caregivers the rationale for measurements of disease severity. Such an approach is consistent with other calls for regulators to generally clarify the rationale for their decisions, and to cite precedent and similar cases as part of their evidentiary support.^11^

## Enhance coordination between key stakeholders

A significant challenge is the lack of coordination among key stakeholders in the AA ecosystem. Life sciences companies have separate conversations with regulators and HTA bodies, a problem compounded by legal constraints on sharing information between agencies in some settings.^12,13^ Payers are typically not part of either conversation, and regulatory/HTA engagement with patients and caregivers is highly variable. One thread of discussion at the Bellberry meeting focused on the benefits of bringing all parties together for scientific dialogue on surrogate and traditional endpoints, registrational and confirmatory trial design, and other issues while the drug is still in early clinical development, although it was noted that some agencies might need increased capacity to offer such services, and data requirements as well as interagency coordination may also be challenging. The US Bipartisan Policy Center recently created a set of recommendations to strengthen coordination between the US FDA and Centers for Medicare and Medicaid Services^14^; some of these synergies may well be feasible across other jurisdictions:

- Quarterly communication from the regulator to the HTA body/payer on new drug applications, applications on an expedited approval pathway, and timing of any advisory or deliberative committee meetings as well as relevant meeting materials
- Legal and regulatory scrutiny of limitations on communication between parties, with granting of waivers as warranted
- Creation of a common warehouse for clinical trial and real-world data that can be shared bi-directionally between agencies
- Allow HTA representatives to observe all discussions between manufacturer and regulator

There may also be clear opportunities for patient and caregiver engagement. Patient advocates could be invited to observe regulator-manufacturer discussions, be representatives to data governance committees that review research protocols and discuss preliminary findings, and be participants in development of lay-friendly materials communicating the potential benefits and risks of a new treatment as well as the level of uncertainty about these outcomes (see “Improving key stakeholder understanding of and support for AA drugs and processes” below).

## Create an accelerated pathway in HTA and payer settings

Health technology assessment agencies, payers, and other decision-makers consistently face a number of challenges with medicines approved through expedited regulatory pathways, including uncontrolled studies, limited duration of follow-up, and smaller sample sizes. However, few, if any, of these organizations appear to have a designated technology appraisal pathway for AA medicines. Creating a distinct accelerated pathway for appraisal would send appropriate signals to manufacturers, patient organizations, and other stakeholders that a unique process is in play that recognizes both urgency of need and inherent uncertainty in evidence. A more rapid timeline for review could be contemplated given the limited evidence available. Additional requirements for manufacturers could be included, such as proposals for outcomes-based contracts, real-world evidence studies to bridge the gap between initial regulatory approval and confirmatory trials, or other risk-mitigation strategies. Early conversations between HTA bodies and manufacturers could be held to discuss the linkage of surrogate endpoints to long-term outcomes in economic modeling, patient subgroups more or less likely to benefit, and other critical concerns. As with regulatory pathways, a distinct HTA/payer AA process would provide guidance on concessions and modifications the decision-making authority will make as well as specific expectations for manufacturers and other stakeholders, both before and after market access is provided. Explicit provisions for restricting or withdrawing reimbursement when confirmatory trials are delayed or negative could also be included, as could provisions for ongoing reimbursement to recognize better-than-expected outcomes (see “Link pricing policy to data uncertainty” below). The initiation of a distinct process would require additional resources; although once established, it could help to streamline current processes. As with regulatory authorities, HTA bodies should have formal authority to modify reimbursement or coverage if real-world drug performance diverges from initial expectations.

## Develop joint guidance on study design and data needs

While the ability of regulators and HTA bodies or payers to offer customized early scientific advice to individual companies will vary across settings, there are other opportunities for these organizations to come together. The FDA has a long history of developing guidance for manufacturers on outcome selection, approaches to measurement, and other study design considerations for interventions in specific disease areas.

Inclusion of HTA agencies or payers in such efforts could broaden the nature of these considerations in an AA setting and streamline clinical development so that pivotal studies are designed to meet the needs of multiple decision-makers.^15^ For example, new anti-cancer medicines approved on an expedited pathway are often studied in patients with later-stage cancer with few to no alternative treatments available. These single-arm studies may lead to expedited approval based on a surrogate endpoint such as tumor response, but then a separate randomized trial must be conducted in an earlier-stage population to confirm benefit. Recent recommendations in oncology have called for both expedited and full approval to be based on the same randomized trial, with expedited approval based on an interim analysis of the surrogate and full approval granted based on longer-term survival data from the same study.^16^ In England, joint guidance focused specifically on early access to promising medicines is available from the regulator, HTA agency, and payer, although this has been more focused on access and less so on evidence generation to date.^17^

Joint guidance could also address data-collection strategies to support accelerated access in the marketplace while awaiting confirmatory trial results. Rigorous studies using electronic health records or administrative datasets could provide early information on issues of concern to both types of agencies, including exploration of safety signals, assessment of durability of benefit and adherence to treatment, and, in some cases, early indications of the longer-term outcomes of interest (eg, cardiovascular events for anti-diabetic therapies, disease progression for anti-cancer medicines). As with other efforts, joint guidance will require additional resources, personnel, and coordination between authorities.

## Link pricing policy to data uncertainty

Because payers are concerned about both clinical and economic risk, and HTA principles support both access and reimbursement, these communities are understandably concerned about risks inherent in paying a premium price for a medicine that ultimately does not prove beneficial. So-called “managed entry” and outcomes-based agreements are increasing in number for therapies approved via expedited pathways; most frequently, the premium price is paid initially with rebate or claw-back features if certain outcomes or milestones are not achieved.^18,19^ This allows economic risk to be shared between the manufacturer and the payer, the exposure of each depending upon the agreed timing of milestones and level of funds to be returned by the manufacturer. While the feasibility of such agreements varies and may depend on data, resources, and relevant regulation and policies in each jurisdiction, interest in their use continues to grow.^20^

Lower expectations for evidence generation and speedier time to market under expedited approval programs result in significant revenue potential for the manufacturer, but the value proposition for payers is uncertain because of the limited evidence available. Integrating that uncertainty into initial pricing, with a commensurate increase in price when uncertainty is reduced,^21^ would allow early revenue to accrue to the manufacturer while further evidence is generated and/or benefit is confirmed, and provide some financial risk protection to the payer. Such an approach was recently endorsed by the current FDA Commissioner.^22^ Regardless, some change in policy is warranted, as current incentives for manufacturers to complete confirmatory studies requested by regulators are limited.^23^

There are a number of ways this can be done. In situations where the level of benefit is completely unclear or unknown, the initial price could be referenced to that of a relevant comparator or a market basket of alternatives. When some level of benefit is suggested, the price estimated by the most conservative scenario of a cost-effectiveness analysis (CEA) or the price that would meet a lower-bound threshold of cost-effectiveness could be used initially. Then, as additional evidence is generated and the CEA model is modified, there will be more confidence in central estimates of economic impact, and likely an increase in the cost-effective price. We note that the use of CEA for such purposes is widely accepted in many but not all jurisdictions (eg, Germany, the United States), so other approaches would be required. Regardless, for any approach to be accepted, the payer must commit to paying a higher price when new evidence justifies it (see “Develop joint guidance on study design and data needs” above).

## Improving key stakeholder understanding of and support for AA drugs and processes

Much of what is described above focuses on “prepping the system” for more orderly handling of medicines in an AA program and coordination between multiple decision-makers. But what of the end-users and beneficiaries of these products: the patients who need them, the clinicians who prescribe them, and society, which sees the health, social, and economic benefits? There is often no clear articulation of the potential benefits and risks of these drugs, the link between the endpoints measured in initial studies and the ultimate outcomes of interest to patients and caregivers, and the plan for confirmation of benefit. Patients, in particular, also need to know what will happen to the medicine if the confirmatory trial fails (this differs by jurisdiction and sometimes even by medicine).^24,25^ There is also limited engagement of patients, clinicians, and the wider public in the development and operation of the various systems that regulators, HTA bodies, and payers have in place for medicines on expedited approval pathways.

There has already been an effort to develop lay-friendly summaries of pivotal clinical studies, one that involves active collaboration between manufacturers and patient advocates.^26,27^ This could be enhanced through participation of regulatory and HTA representatives, with additional information on actual or expected validation of surrogate endpoints, descriptions of how known risks have been or will be managed, key uncertainties in the evidence available, and any plans to collect real-world data to complement the confirmation process. A draft of the summary should be made publicly available as soon as the medicine is accessible—after regulatory approval in the United States, but typically after the HTA appraisal elsewhere.

In addition, regulators could be required to publish and invite comment on draft decisions—with associated reasoning and justification—on granting expedited status to medicines and the associated designs for initial and confirmatory studies. Similarly, HTA bodies and payers could be required to publish and invite comment on draft decisions—with associated reasoning and justification—on coverage and pricing agreements and associated post-market studies. This could help increase public confidence in and support for the systems they operate and the decisions they make.

## Conclusion

A coordinated AA ecosystem will not be built overnight. There are many open questions regarding governance, the levels of intra-national and international cooperation required, and sources of funding for additional data collection, dedicated personnel, and development of materials, among others. But making a start on the actions we have described is entirely feasible, as the focus is on putting additional detail into principles already used, getting people already doing the work to talk to one another, and transparently documenting the approaches already being taken. The twin goals of speedy access and sound evidence need not be at odds if the process is coordinated, and all parties understand their roles in it.

## Supplementary Material

qxae095_Supplementary_Data

## References

[1] Franco P , Jain R, Rosenkrands-Lange E, Hey C, Koban MU. Regulatory pathways supporting expedited drug development and approval in ICH member countries. Ther Innov Regul Sci. 2023;57(3):484–514. 10.1007/s43441-022-00480-3 36463352 PMC9734413

[2] Bellberry Ltd . Our company. Accessed May 30, 2024. https://bellberry.com.au/about-us/our-company/

[3] Bellberry Ltd . Towards a coordinated approach for managing accelerated patient access to potentially beneficial medicines: balancing patient, regulator, HTA, payer, and other stakeholder needs. April 2024. Accessed August 08, 2024. https://bellberry.com.au/wp-content/uploads/BbL-SYMPOSIUM-REPORT.pdf

[4] Bellberry Ltd . Meeting statement: international scientific congress. April 2024. Accessed August 08, 2024. https://bellberry.com.au/wp-content/uploads/23-327-BbL-DOCUMENT-Symposium23-Statement-2_27NOV2023.pdf

[5] Phillips M , Synnott P, Henshall C, Tunis S, Sansom L, Ollendorf D. Towards a coordinated approach for managing accelerated patient access to potentially beneficial medicines: reporting the perspectives of a multi-stakeholder, international workshop. Health Aff Scholar. 2024;2(6):qxae069. 10.1093/haschl/qxae069 PMC11157170 38855055

[6] Kepplinger EE . FDA's expedited approval mechanisms for new drug products. Biotechnol Law Rep. 2015;34(1):15–37. 10.1089/blr.2015.9999 25713472 PMC4326266

[7] Bloem LT , Schelhaas J, López-Anglada L, Herberts C, van Hennik PB, Tenhunen O. European conditional marketing authorization in a rapidly evolving treatment landscape: a comprehensive study of anticancer medicinal products in 2006-2020. Clin Pharmacol Ther. 2023;114(1):148–160. 10.1002/cpt.2906 37129347

[8] Murray CJL . The Global Burden of Disease Study at 30 years. Nat Med. 2022;28(10):2019–2026. 10.1038/s41591-022-01990-1 36216939

[9] Mahdiani H , Münch N, Paul NW. A QALY is [still] a QALY is [still] a QALY? Evaluating proportional shortfall as the answer to the problem of equity in healthcare allocations. BMC Med Ethics. 2024;25(1):35. 10.1186/s12910-024-01036-w 38521941 PMC10960401

[10] Ciani O , Buyse M, Drummond M, Rasi G, Saad ED, Taylor RS. Time to review the role of surrogate end points in health policy: state of the art and the way forward. Value Health. 2017;20(3):487–495. 10.1016/j.jval.2016.10.011 28292495

[11] Janiaud P , Irony T, Russek-Cohen E, Goodman SN. U.S. Food and Drug Administration reasoning in approval decisions when efficacy evidence is borderline, 2013-2018. Ann Intern Med. 2021;174(11):1603–1611. 10.7326/M21-2918 34543584

[12] Burgess N , Currie G, Crump B, Richmond J, Johnson M. Improving together: collaboration needs to start with regulators. BMJ. 2019;367:l6392. 10.1136/bmj.l6392 31776111 PMC6880245

[13] Center for Innovation in Regulatory Science. August 2021. Accessed April 1, 2024. https://cirsci.org/wp-content/uploads/dlm_uploads/2022/01/CIRS-March-2021-workshop-report-Interactions-and-collaborations.pdf

[14] Bipartisan Policy Center. January 2024. Accessed April 1, 2024. https://bipartisanpolicy.org/download/? file=/wp-content/uploads/2023/12/CMS-FDA-Final-Report.pdf

[15] Iorio A , Skinner MW, Clearfield E, et al Core outcome set for gene therapy in haemophilia: results of the coreHEM multistakeholder project. Haemophilia. 2018;24(4):e167–e172. 10.1111/hae.13504 29781145

[16] Fashoyin-Aje LA , Mehta GU, Beaver JA, Pazdur R. The on- and off-ramps of oncology accelerated approval. N Engl J Med. 2022;387(16):1439–1442. 10.1056/NEJMp2208954 36129992

[17] NHS England . Early access to medicines scheme. Accessed April 1, 2024. https://www.england.nhs.uk/aac/what-we-do/how-can-the-aac-help-me/eams/

[18] Zampirolli Dias C , Godman B, Gargano LP, et al Integrative review of managed entry agreements: chances and limitations. Pharmacoeconomics. 2020;38(11):1165–1185. 10.1007/s40273-020-00943-1 32734573

[19] Ispán F , Hegedüs T, Csanádi M, Nagy B. Goals and methods of managed entry agreements—can we get what we want? Health Policy Technol. 2023;12(2):100745. 10.1016/j.hlpt.2023.100745

[20] Huang D , Mohammed R. Exploring the trend of value-based contracts in the United States. forHealth Consulting at UMass Chan Medical School. July 2024. Accessed February 27, 2023. https://forhealthconsulting.umassmed.edu/exploring-the-trend-of-value-based-contracts-in-the-united-states/

[21] Robinson JC . Drug pricing with evidence development. JAMA. 2022;327(16):1545–1546. 10.1001/jama.2022.5403 35404379

[22] Weisman R. “FDA chief's unwelcome message to biotech execs: ‘Prices of drugs are too high’.” *Boston Globe*. April 2024. Accessed June 7, 2023. https://www.bostonglobe.com/2023/06/07/business/fda-chiefs-unwelcome-message-biotech-execs-prices-drugs-are-too-high/

[23] Frank RG , Shahzad M, Emanuel EJ. Accelerated approval of cancer drugs: no economic reward for drug makers that conduct confirmatory trials. Health Aff (Millwood). 2022;41(9):1273–1280. 10.1377/hlthaff.2022.00119 35977352

[24] Gyawali B , Rome BN, Kesselheim AS. Regulatory and clinical consequences of negative confirmatory trials of accelerated approval cancer drugs: retrospective observational study. BMJ. 2021;374:n1959. 10.1136/bmj.n1959 34497044 PMC8424519

[25] Van Norman GA . Drugs and devices: comparison of European and U.S. approval processes. JACC Basic Transl Sci. 2016;1(5):399–412. 10.1016/j.jacbts.2016.06.003 30167527 PMC6113412

[26] Health Technology Assessment International . Summary of Information for Patients (SIP) Project. Accessed April 1, 2024. https://past.htai.org/summary-of-information-for-patients-project/.

[27] National Institute for Health and Care Excellence . Summary of Information for Patients (SIP): international SIP template. Accessed April 1, 2024. https://www.nice.org.uk/guidance/ta959/documents/committee-papers-2

